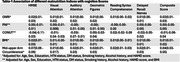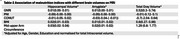# Nutritional Deficits and their Impact on Brain Morphology and Cognition in a Southern Indian Rural Community

**DOI:** 10.1002/alz70857_106650

**Published:** 2025-12-26

**Authors:** Sulakshna Aggarwal, Priya Chatterjee, Hitesh Pradhan, Raghav Prasad, Jonas S Sundarakumar, Centre for Brain Research‐ Srinivaspura Aging Neurosenescence and Cognition

**Affiliations:** ^1^ Centre for Brain Research, Bangalore, Karnataka, India; ^2^ Centre for Brain Research, Indian Institute of Science, Bangalore, Karnataka, India; ^3^ Centre for Brain Research, Indian Institute of Science, Bangalore, India

## Abstract

**Background:**

Malnutrition is associated with poorer cognitive performance, though the evidence is predominately from Caucasian and urban populations. We aim to study the association between malnutrition, cognitive performance, and brain morphology in a rural southern Indian population.

**Method:**

We used baseline cross‐sectional data from the Centre for Brain Research‐Srinivaspura Aging NeuroSenescence and COGnition (CBR‐SANSCOG) study cohort of non‐demented participants aged 45+ years (*n* = 3687). Malnutrition was assessed using multivariable indices‐ Geriatric Nutritional Risk Index (GNRI), Prognostic Nutritional Index (PNI), CONUT (Controlling Nutritional Status) score, and anthropometric indices‐ body mass index (BMI) and mid‐upper arm circumference (MUAC). We assessed the agreement of each nutritional index with the Standard Combined Index (SCI). Cognitive performance was assessed using Hindi Mental Status Examination (HMSE) and culturally adapted COGNITO test battery (Computerized assessment of adult information processing). The domain‐specific (attention, memory, language, visuospatial) cognitive scores were standardized and a composite score was calculated from the weighted averages of the individual test scores. General linear model was used to assess the association between the malnutrition indices and cognitive performance, adjusting for age, sex, education, tobacco, alcohol use, hypertension, diabetes, and depression. In a subset of participants who underwent 3T MRI (*n* = 1150), region of interest (ROI)‐based analysis was done to study the association between the malnutrition indices and brain ROI volumes normalized for total intracranial volume, adjusting for age, sex, and education.

**Result:**

Based on MUAC, GNRI, PNI, and CONUT score, 8.8%, 9%, 15%, and 19% of the participants (mean age: 57.49±9 years) were at a risk of malnutrition, respectively. GNRI had the best agreement with the SCI. We found a positive association between GNRI, BMI, MUAC scores and cognitive performance (Table 1) and ROI brain volumes (Table 2). MUAC had a stronger relationship to cognitive domains than BMI. No association was observed between PNI and CONUT scores and any of the outcome variables.

**Conclusion:**

GNRI, a multivariable malnutrition index is a reliable predictor of cognitive performance and brain morphology. Additionally, MUAC, a simple and cost‐effective anthropometric measure, could be a potential indicator of rural Indians at risk of cognitive impairment.